# Inhibition of Mammalian Target of Rapamycin in Human Acute Myeloid Leukemia Cells Has Diverse Effects That Depend on the Environmental *In Vitro* Stress

**DOI:** 10.1155/2012/329061

**Published:** 2012-10-02

**Authors:** Anita Ryningen, Håkon Reikvam, Ina Nepstad, Kristin Paulsen Rye, Øystein Bruserud

**Affiliations:** ^1^Division of Hematology, Institute of Medicine, University of Bergen, N-5021 Bergen, Norway; ^2^Department of Medicine, Haukeland University Hospital, N-5021 Bergen, Norway

## Abstract

Effects of the mTOR inhibitor rapamycin were characterized on *in vitro* cultured primary human acute myeloid leukemia (AML) cells and five AML cell lines. Constitutive mTOR activation seemed to be a general characteristic of primary AML cells. Increased cellular stress induced by serum deprivation increased both mTOR signaling, lysosomal acidity, and *in vitro* apoptosis, where lysosomal acidity/apoptosis were independent of increased mTOR signaling. Rapamycin had antiproliferative and proapoptotic effects only for a subset of patients. Proapoptotic effect was detected for AML cell lines only in the presence of serum. Combination of rapamycin with valproic acid, all-trans retinoic acid (ATRA), and NF-**κ**B inhibitors showed no interference with constitutive mTOR activation and mTOR inhibitory effect of rapamycin and no additional proapoptotic effect compared to rapamycin alone. In contrast, dual inhibition of the PI3K-Akt-mTOR pathway by rapamycin plus a PI3K inhibitor induced new functional effects that did not simply reflect a summary of single drug effects. To conclude, (i) pharmacological characterization of PI3K-Akt-mTOR inhibitors requires carefully standardized experimental models, (ii) rapamycin effects differ between patients, and (iii) combined targeting of different steps in this pathway should be further investigated whereas combination of rapamycin with valproic acid, ATRA, or NF-**κ**B inhibitors seems less promising.

## 1. Introduction

Acute myeloid leukemia (AML) is a heterogeneous malignancy characterized by bone marrow infiltration of immature leukemic myeloblasts, and the overall disease-free survival is only 40–50% even for the younger patients below 60–65 years of age who receive the most intensive chemotherapy [1, 2]. New therapeutic approaches are thus warranted [3], and inhibition of the phosphatidylinositol 3-kinase (PI3K)-Akt-mammalian target of rapamycin (mTOR) pathway may become a future strategy because this pathway is constitutively activated in the leukemia cells for most patients and seems important for regulation of cell proliferation, viability, and autophagy [4–8]. However, despite these observations the initial clinical studies showed an antileukemic effect of mTOR inhibition only for a subset of patients [9]. Thus, the future development and optimal use of PI3K-Akt-mTOR inhibition as a therapeutic strategy in human AML will probably depend on a more detailed functional characterization of this pathway using standardized *in vitro* models [4–7]. 

## 2. Material and Methods

### 2.1. Pharmacological Agents

The first generation mTOR inhibitor rapamycin was purchased from LC Laboratories (Woburn, MA, USA). The PI3K inhibitor 3-methyladenine (3-MA) and the specific I*κ*B-kinase/NF*κ*B inhibitor BMS-345541 were purchased from Sigma Aldrich (St. Louis, MO, USA). Stock solutions were dissolved in dimethylsulphoxide (DMSO), aliquoted, and stored at −80°C. The stock solutions were further diluted in culture medium. Pilot experiments showed that DMSO at concentrations used in the experiments did not affect AML cell proliferation. Valproic acid was from Orfiril; Destin GmbH (Hamburg, Germany) and aliquoted stock solutions in saline were stored at −80°C and further diluted with culture medium. The HSP90 inhibitor 17-dimethylaminoethylamino-17-demethoxygeldanamycin (17-DMAG) was purchased from Infinity Pharmaceuticals (Cambridge, MA, US) and used at 1.0 *μ*M.

### 2.2. AML Cell Cultures

The study was approved by the local Ethics Committee (University of Bergen, Norway) and patient samples collected after written informed consent. The study included primary human AML cells from unselected adult patients with peripheral blood blast counts exceeding >7 × 10^9^/L and being >80% of the circulating leukocytes. The AML cell lines HL60, HEL, K562, KG1a, and CTV-1 and the acute lymphoblastic leukemia (ALL) cell lines Nalm-6 and Tanoue were purchased from Deutsche Sammlung von Mikroorganismen und Zellkulturen GmbH (GSMZ; Braunschweig, Germany). The culture medium was Stem Span (Stem Cell Technologies, Vancouver, BC, Canada) eventually supplemented with 10% heat-inactivated fetal bovine serum (FBS) [10]. Primary AML cells were isolated from the blood by density gradient separation (Lymphoprep, Axis-Shield, Oslo, Norway), contained at least 95% leukemia blasts [11, 12] and were stored in liquid nitrogen [11, 12]. 

### 2.3. Analysis of Viability, Proliferation, and Flow Cytometry

#### 2.3.1. Viability

Leukemic cells (2 × 10^6^ cells in 2 mL) were incubated at 37°C in a humidified atmosphere of 5% CO_2_ in 24-well culture plates (Costar 3524; Cambridge, MA, USA) for 48 hours in StemSpan SFEM medium (referred to as StemSpan; Stem Cell Technologies; Vancouver, BC, Canada) supplemented with 100 *μ*g/mL of gentamicin. The fractions of viable, apoptotic, and necrotic cells were then determined by double staining of AML cells with Annexin V-fluorescein isothiocyanate and propidium iodide (PI) (Apoptest-FITC kit; NeXins Research, Kattendijke, the Netherlands) as described in detail previously [13]. 

#### 2.3.2. Proliferation

AML cells 5 × 10^4^/well were cultured in 150 *μ*L medium in flat-bottomed microtiter 96-well plates (Nucleon Surface, Nunc A/S, Roskilde, Denmark). Cells were cultured in medium alone or with stem cell factor (SCF), granulocyte-macrophage colony stimulating factor (GM-CSF) and FLT3 ligand (FLT3-L) (all from PeproTech Ltd.; Rocky Hill, NJ, USA). Nuclear ^3^H-thymidine incorporation was assayed after seven days as described in detail previously [14].

#### 2.3.3. Flow Cytometry

Cultured cells were washed with phosphate buffered saline (PBS) and fixed with 4% paraformaldehyde (PFA) in PBS before permeabilization with ice-cold methanol. After washing twice with PBS samples were blocked with 5% bovine serum albumin (BSA) in PBS before being incubated with primary conjugated fluorescent antibodies against phospho-S6RP (S6 ribosomal protein) and the autophagy-associated mediators LC3B and Beclin-1 (Cell Signaling Technology, Inc.; Boston, MA, USA) and ATG-3, ATG-7, ATG-10 (Biosensis; Halifax, Australia) for 1 hour. After PBS washing samples were analyzed by flow cytometry. The mean fluorescence intensity (MFI) was detected for the cells after eliminating debris and cell aggregates in a forward versus side scatter cytogram.

Lysosomal acidity was detected with LysoTracker Red DND-99 from Molecular Probes, Inc. (Eugene, OR, USA). Aliquots of the 1 mM probe stock solution were stored at −20°C and diluted to a final concentration of 50 nM in growth medium. The cells were incubated with LysoTracker Red for the last 30 minutes of culture, washed with PBS, fixed with 4% paraformaldehyde (PFA) in PBS, and washed with PBS before being analyzed by flow cytometry. A cytogram based on forward versus side scatter was used to eliminate debris and cell aggregates before lysosomal acidity was analyzed.

For the cell cycle measurements PI and RNase A were purchased from Sigma-Aldrich. The cells were rapidly fixed in ice-cold 70% ethanol and incubated for at least 30 minutes at 4°C. They were then washed once with PBS, resuspended in 800 *μ*L PBS + RNase A (0.1 mg/mL) + PI (40 *μ*g/mL) before cells were incubated in the dark at 37°C for 30 minutes. Samples were immediately analyzed by flow cytometry. A cytogram based on forward versus side scatter was used to eliminate aggregates, debris, and dead cells before red fluorescence in linear mode was detected on viable cells.

### 2.4. Statistical and Bioinformatical Approaches

All statistical analyses were performed using the Statistical Package for the Social Sciences (SPSS) version 15.0 (SPSS Inc., Chicago, IL, USA) and GraphPad Prism 5 (GraphPad Software, Inc., San Diego, CA, USA), and *P* values < 0.05 were regarded as statistically significant. Bioinformatical analyses were performed using the J-Express 2011 analysis suite (MolMine AS, Bergen, Norway) [15, 16]. Values were divided by the values of control culture before being transformed to logarithmic values (base 2) as described previously [16]. Unsupervised hierarchical clustering was performed with Euclidian correlation and complete linkage as distance measure.

## 3. Results

### 3.1. Primary Human AML Cells Show Constitutive mTOR-Mediated Signaling and a Wide Variation in the Expression of Proteins Involved in Autophagy

**Figure 1:**
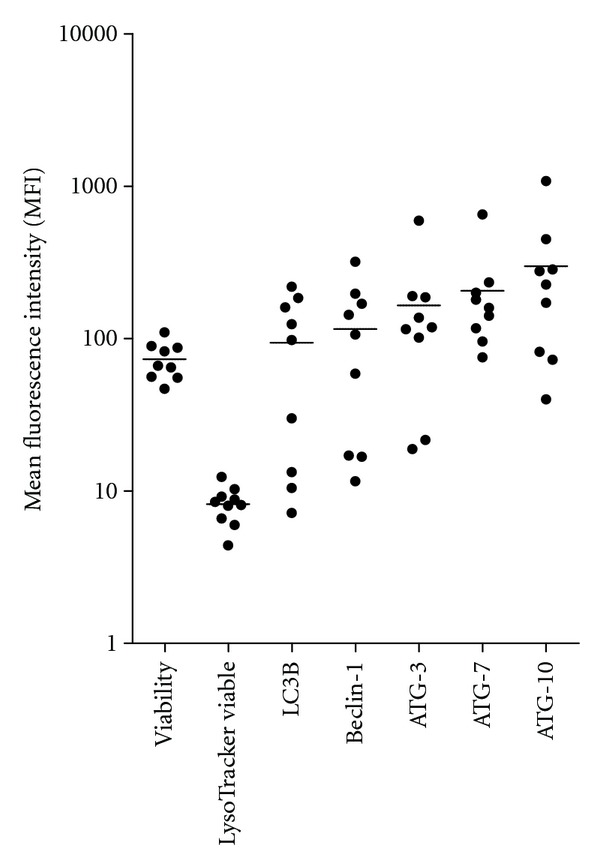
Intracellular levels of the five autophagy-involved mediators LC3B, Beclin-1, ATG3, ATG7, and ATG10 in primary human AML cells. Levels were determined by flow cytometric analysis for primary human AML cells derived from 9 unselected patients. The cells were rested in FCS-containing medium for 4 hours before the analysis. The results are presented as the mean fluorescence intensity (MFI).

We compared the intracellular levels of the phosphorylated mTOR target S6RP and the autophagy-associated mediators LC3B, Beclin-1, ATG-3, ATG7, and ATG-10 after 4 hours of incubation in FBS-containing medium for AML cells derived from 9 patients (Figure 1). Constitutive signaling through mTOR was estimated as the MFI of phosphorylated S6RP (p-S6RP); this was detected for all patients but the levels showed a wide variation (MFI range 50–405). Expression of LC3B (MFI range 16.2–65.3), Beclin-1 (range 9.4–101.5), ATG-3 (range 28.5–116.3), ATG7 (range 31.2–118.5), and ATG-10 (range 9.1–105.6) also showed wide variations without any correlation with S6RP phosphorylation.

**Figure 2:**
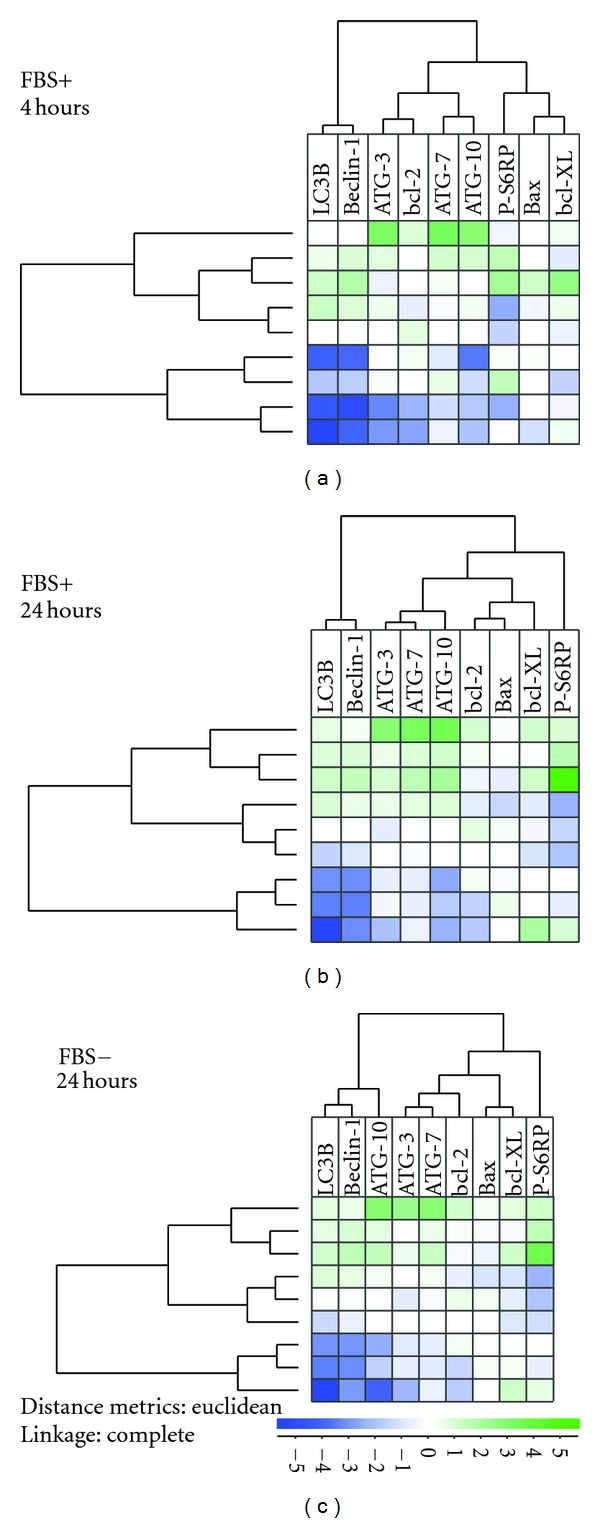
Unsupervised hierarchical cluster analysis of the intracellular levels of (i) the five autophagy-involved mediators LC3B, Beclin-1, ATG3, ATG7 and ATG10, (ii) the apoptosis regulators bcl-2, bcl-XL, and bax, and (iii) the phosphorylated form of the mTOR downstream target S6RP. The levels were determined by flow cytometry for primary human AML cells derived from 9 unselected patients. Cells were incubated in either FBS-containing or serum-free medium for 4 and 24 hours before analysis. The figure presents the results for cells incubated in FBS-containing medium for 4 (a) and 24 hours (b) and for cells incubated in serum-free medium for 24 hours (c).

We did an unsupervised hierarchical clustering of the patients with regard to levels of autophagy-associated molecules (Figure 2). The patient clustering showed only minor differences between FBS-containing and serum-free cultures, and as would be expected from the correlation analyses (see above) the p-S6RP level clustered separately with no close association with any of the autophagy mediators. Both FBS-containing and serum-free cultures showed close associations between (i) LC3B and Beclin-1; (ii) the three ATGs, and (iii) apoptosis-regulating bcl-2, bcl-Xl and bax. 

### 3.2. Dose-Response Effects of Rapamycin on Primary Human AML Cell Proliferation

We investigated the effect of different rapamycin concentrations (tenfold dilution between 0.01 nM and 10^5^ nM) on cytokine-dependent AML cell proliferation for 15 unselected patients. All concentrations caused a similar and statistically significant inhibition of AML cell proliferation with median proliferation varying between 68% (0.01 and 10^4^ nM) and 77% (10^3^ nM). Studies of myeloma cells have also described a similar antiproliferative effect of different concentrations of rapamycin when tested over a wide concentration range [17], and previous *in vitro* studies of primary human AML cells suggest that some patients show no inhibition of mTOR activity when testing rapamycin ≤20 nM and with a maximal effect being reached at rapamycin >50 nM [18]. Based on our own dose-response experiments and these previous observations we used rapamycin 100 nM in our experiments.

### 3.3. Rapamycin-Induced mTOR Inhibition Does Not Reverse the Stress-Induced Increase in Lysosomal Acidity and Spontaneous Apoptosis in Primary Human AML Cells

Even *in vitro* culture in optimal FBS-containing medium is associated with spontaneous apoptosis of primary human AML cells as well as a small but significant increase in lysosomal acidity (see Supplementary Figure 1(a) available online at doi:10.1155/2012/329061). Serum deprivation during culture is often used to increase cellular stress [19–24], and for primary AML cells such deprivation is associated both with a further increase in spontaneous apoptosis (Supplementary Figure 1(b)) and in addition increased mTOR signaling and increased lysosomal acidity (Supplementary Figures 1(c) and 1(d)) even though the intracellular levels of autophagy- (LC3B, Beclin, ATG3, ATG7, ATG10) or apoptosis-associated (bcl-2, bcl-XL, bax) molecules are not altered.

**Figure 3:**
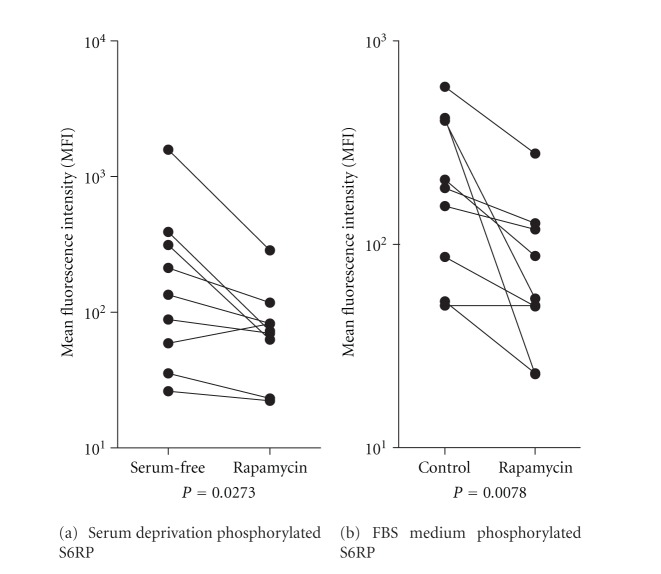
Effects of *in vitro* culture of primary human AML cells on mTOR signaling for primary human AML cells derived from unselected patients. The figure compares mTOR-mediated signaling (levels of the downstream target p-S6RP) for AML cells incubated for 24 hours in serum-free (a) or FBS-containing (b) medium alone or in the presence of rapamycin 100 nM. The results are presented as the median fluorescence intensity (MFI).

Rapamycin 100 nM significantly reduced phosphorylation of the mTOR downstream target S6RP when AML cells were cultured under serum-free conditions; this decrease was detected after only 4 hours (Figure 3; *P *= 0.008) and persisted after 24 hours (*P* = 0.028, data not shown). However, rapamycin 100 nM (24 hours cultures) had divergent effects and caused no significant alterations when comparing the overall results for cell viability, lysosomal acidity, or intracellular levels of autophagy-associated and apoptosis-regulating molecules (data not shown). Rapamycin did not affect apoptosis or lysosomal acidity for primary AML cells cultured under optimal conditions with FBS-containing medium either (data not shown) even though it decreased S6RP phosphorylation (Figure 3). These results demonstrate that the decreased viability and increased lysosomal acidity induced by serum deprivation (i.e., experimental *in vitro* stress) are not reversed by inhibition of the increased mTOR signaling. Thus, spontaneous or stress-induced *in vitro* apoptosis and mTOR signaling seem to be independent events. 

As described previously the extent of spontaneous *in vitro* apoptosis shows wide variation between patients both in the presence of rapamycin 100 nM and in drug-free cultures. Furthermore, the cellular HSP70/HSP90 levels then seem important for the extent of this apoptosis [13]. We therefore investigated whether the patient heterogeneity could be reduced by adding the HSP90 inhibitor 17-DMAG 50 nM and thereby reducing the influence of variations in intracellular HSP90 levels between patients. We examined AML cells from 52 patients, but the patient heterogeneity with regard to the effect of rapamycin on cell viability persisted also in the presence of 17-DMAG. Thus, the patient heterogeneity with regard to effect of rapamycin on primary AML cell viability is not secondary to differences in HSP90 levels.

### 3.4. Rapamycin Has a Proapoptotic Effect on AML Cell Lines Associated with Increased Lysosomal Acidity, but This Effect Is Not Detected during Serum Deprivation

As described previously primary human AML cells show spontaneous or stress-induced *in vitro* apoptosis during *in vitro* culture, and this is seen especially during serum depletion but also for cultures supplemented with FBS. We therefore used an alternative experimental model and investigated effects of rapamycin on the viability of the five AML cell lines HL60, HEL, KG1a, CTV-1, and K562. All these cell lines proliferated when cultured in serum-free medium [13, 25], but the viability was generally lower in serum-free than FBS-containing cultures. We investigated the effect of rapamycin (100, 200, 400, 600, and 800 nM) when AML cells were cultured in medium without and with 10% inactivated FBS. For these cell lines rapamycin caused a dose-dependent reduction in AML cell viability in FBS-containing cultures. In contrast, for the serum-free cultures rapamycin even caused a minor increase in the viability for HEL (rapamycin 100 nm, viability 63 versus 76%), HL60 (81 versus 87%), and CTV-1 cells (78 versus 89%); for higher concentrations the increased viability was also observed for K562 (600 nM, 59 versus 77% viable cells). The results for HEL and CTV-1 are presented in Supplementary Figure 2. The FBS dependency of this rapamycin-associated proapoptotic effect was reproduced for all cell lines in 4 independent experiments, the proapoptotic effect in FBS-containing cultures could be detected after 24 hours and increased gradually during the first 48 hours of culture, and the early apoptotic population remained small during culture but showed a minor increase for all except K562 (data not shown). Finally, we observed a decrease in the phosphorylation of the mTOR downstream target S6RP in all rapamycin-supplemented cultures whether FBS was added or not. To conclude, (i) AML cell lines cultured in medium alone show detectable stress-induced *in vitro* apoptosis similar to primary cells, and rapamycin then does not alter regulation of apoptosis, whereas (ii) cell lines cultured in FBS containing medium show very low stress-induced apoptosis, and under these conditions rapamycin has a proapoptotic effect.

We investigated the lysosomal acidity for all AML cell lines and the ALL cell line Tanoue when cells were cultured with and without rapamycin 100 nM in serum-free and FBS-supplemented medium. The lysosomal acidity/autophagy in drug-free controls was always highest for cells cultured without FBS, and a similar difference between serum-free and FBS containing cultures was seen even in the presence of rapamycin (Supplementary Figure 3), but without any significant effect of rapamycin on autophagy (data not shown). These results were reproduced in independent experiments for all cell lines. Thus, similar to the primary cells serum deprivation of AML cell lines increased lysosomal acidity, and we did not detect any further effect of rapamycin-induced mTOR inhibition on the acidity, that is, autophagy.

### 3.5. Rapamycin Has a Caspase-Independent Proapoptotic Effect in AML Cell Lines

We compared cell viability in FBS-containing cultures prepared with rapamycin alone and rapamycin plus the pan-caspase inhibitor Z-VAD for three AML cell lines. For KG1a and K562 the viability was not increased by caspase inhibition. For HL60 the viability after 24 hours was 71% in control cultures, it was decreased to 33.1% by rapamycin alone, and a minor increase to 46% was seen when Z-VAD was added together with rapamycin. Thus, caspase-independent mechanisms are important for the rapamycin-induced decrease of AML cell viability, whereas bax and Bcl-2 showed only minor alteration after exposure to rapamycin and/or Z-VAD (only KG1a being examined).

### 3.6. Combined mTOR and PI3K Inhibition: More than a Summary of Single Drug Effects

Other inhibitors of the PI3K-Akt-mTOR pathway are now being developed, including PI3K inhibitors acting upstream to mTOR [26, 27]. These upstream inhibitors are also considered for cancer therapy [26, 27], and we therefore investigated whether combination of rapamycin with an upstream inhibitor had any additional effects compared with rapamycin alone. 3-MA is a paninhibitor of PI3K and is regarded as an inhibitor of autophagy [13]. The effects of 3-MA on KG-1a alone were decreased viability, no effect on lysosomal acidity but increased accumulation of cells in the G0/G1 phase (data not shown). When combined with rapamycin 3-MA caused an additional decrease in viability, but in contrast to the results for 3-MA alone the drug caused increased lysosomal acidity with no additional effect on the G0/G1 fraction when combined with rapamycin. 

### 3.7. Rapamycin Combined with Valproic Acid and ATRA: Valproic Acid Alters Levels of Autophagy-Regulating Mediators in Primary Human AML Cells Whereas ATRA Has Minor Effects

**Figure 4:**
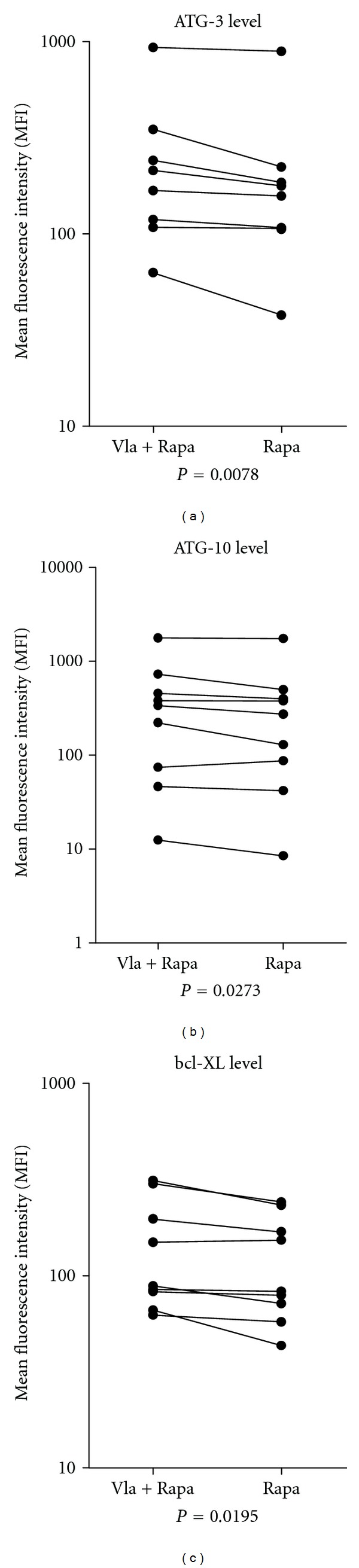
The effect of the HDAC inhibitor valproic acid on the expression ATG-3 (a), ATG-10 (b) and bcl-XL (c) when primary human on AML cells from unselected patients were cultured in serum-free medium in the presence of rapamycin 100 nM alone or in combination with valproic acid (2400 *μ*M). The results are presented as the MFI, and the *P* values are given below each figure. Abbreviations: Vla, valproic acid; Rapa, rapamycin.

Valproic acid plus ATRA is now used for AML-stabilizing palliative therapy either alone or in combination with other drugs [28, 29]. We investigated the effects of combining valproic acid and rapamycin on primary human AML cells (*n* = 9) cultured under optimal *in vitro* conditions in FBS-containing medium. Intracellular levels of ATG-3, ATG-10, and bcl-XL were significantly increased after 24 hours of exposure to the drug combination compared with rapamycin alone (Figure 4) but despite these molecular effects lysosomal acidity (i.e., autophagy) was not altered (data not shown). Valproic acid did not interfere with mTOR-mediated signaling as the phosphorylation status of S6RP in primary AML cells as well as cell lines was not altered when valproic acid was added to rapamycin. Finally, valproic acid plus rapamycin had divergent effects compared with rapamycin alone and when analyzing the overall results the combination did not significantly alter AML cell viability or intracellular levels of bcl-2 or bax (data not shown).

The effect of combining rapamycin and valproic acid on lysosomal acidity and viability was also investigated for all 5 AML cell lines, and similar to the primary cells valproic acid had (i) no or minor effects on lysosomal acidity, the only exception being HEL cells that showed increased lysosomal acidity, and (ii) minor effects on AML cell line viability (data not shown).

ATRA was also tested alone and in combination with rapamycin during 4 and 24 hours of culture for primary AML cells derived from 9 patients. The drug did not interfere with mTOR mediated signaling (i.e. S6RP phosphorylation) and had no additional effect to rapamycin alone on viability, lysosomal acidity, or intracellular protein levels of autophagy-associated (LC3B, Beclin-1, ATG-3, ATG7, ATG-10) and apoptosis-regulating intracellular mediators (bcl-2, bax, bcl-XL) (data not shown). 

### 3.8. Rapamycin Combined with the NF*κ*B Inhibitor BMS-345541 Increases Lysosomal Acidity

The I*κ*B/NF*κ*B inhibitor BMS-345541 was tested alone and in combination with rapamycin during 4 and 24 hours of culture (AML cells from 9 patients examined), but the drug did not have any statistically significant effect on the overall results neither when tested alone nor in combination with rapamycin on the phosphorylation of the mTOR substrate S6RP, viability/apoptosis, lysosomal acidity, or intracellular protein levels of autophagy-associated (LC3B, Beclin-1, ATG-3, ATG7, ATG-10) and apoptosis-regulating intracellular mediators (bcl-2, bax, bcl-XL) (data not shown).

**Figure 5:**
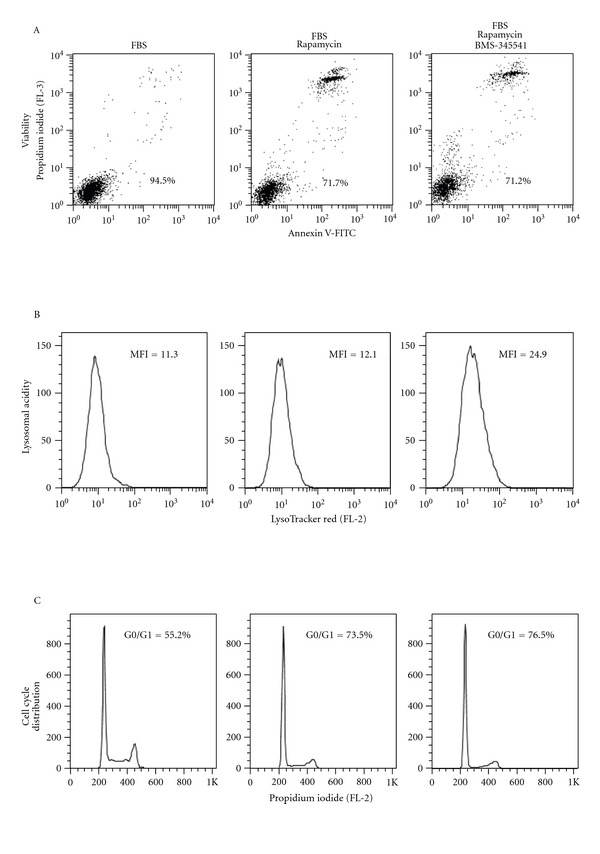
The effect of rapamycin and BMS-345541 on viability (A), lysosomal acidity (B), and cell cycle distribution (C) for the AML cell line KG1a. As is indicated at the top of the figure the AML cells were cultured for 24 hours in FBS-containing medium alone and medium supplemented with rapamycin 100 nM alone or rapamycin plus BMS-345541. The figure presents the results from flow-cytometric analyses of viability ((A) percent of viable cells is given in the diagrams), lysosomal acidity ((B) the MFI values are given in the diagrams), and cell cycle distribution ((C) percent of cells in G0/G1 phase is given in the diagrams).

We investigated the effect of BMS-345541 on the KG1a AML cell line cultured in FBS-containing medium. NF-*κ*B inhibition gradually decreased the AML cell viability during a 48-hour culture period, but the most striking effect was a considerable increase in lysosomal acidity both for viable, apoptotic, and necrotic cells (Figure 5). Furthermore, similar effects were seen when BMS-345541 was added together with rapamycin; the presence of the NF-*κ*B inhibitor then further decreased AML cell viability and caused a gradual and strong increase in lysosomal acidity with maintenance of the rapamycin-induced accumulation of cells in the G0/1 phase during a 48-hour culture period. The increased lysosomal acidity was strongest for the small minority of dead cells but was also seen for viable cells. For viable cells cultured with FBS for 24 hours rapamycin alone increased the MFI of lysosomal acidity to 164% of the drug-free control, whereas an additional increase up to 205% was seen when BMS-345541 was added together with rapamycin. The BMS-345541-associated increase in lysosomal acidity was reproduced in three independent experiments.

To conclude, rapamycin and rapamycin plus BMS-345541 had divergent effects on the viability of primary AML cell viability, but the studies in KG1a cells suggest that decreased viability for a subset of patients is associated with increased autophagy and G0/G1 accumulation of the cells. 

## 4. Discussion

The median age of patients with newly diagnosed AML is 65–70 years. The majority of AML patients thus cannot receive the most intensive and potentially curative treatments for their disease [1, 2], but even for the younger patients receiving this treatment the overall disease-free survival is less than 50% [1, 2]. New therapeutic approaches are therefore considered both for combination with conventional treatment and as a basis for the development of less toxic therapeutic strategies in elderly patients [3]. Inhibition of mTOR-mediated signaling is one of these strategies [4–8], but the initial clinical experience suggests that mTOR inhibitors alone have limited antileukemic effects [9]. Combination of mTOR inhibitors with other targeted therapies should therefore be considered, and preclinical evaluation in well-characterized experimental models will then be important.

Spontaneous or stress-induced apoptosis is seen during *in vitro* culture of primary human AML cells and is most extensive in leukemic cells with low HSP levels as well as a low bcl-2 : bax ratio [13]. Our present results show that this apoptosis is increased during serum deprivation in primary AML cells, and only experimental models using culture of AML cell lines in FBS-containing medium represent a standardized model with a minimal stress-induced apoptosis during culture [10]. The AML cell line viability was also lower during serum deprivation, that is, during experimental *in vitro* stress.

The degree of spontaneous apoptosis differed between culture conditions (serum-deprivation versus FBS-containing medium) and the type of AML cells examined (primary cells versus cell lines). The viability was highest for AML cell lines cultured in FBS-containing medium followed by cell lines cultured in serum-free medium; it was lower for primary AML cells cultured with serum, and the lowest viability was seen for primary AML cells after serum deprivation. Thus, for studies of pharmacological effects without the influence of additional stress-induced apoptosis one should investigate AML cell lines cultured in FBS-supplemented medium, whereas serum-free cultures of primary leukemic cells represent an experimental model with a relatively strong influence of environmental stress-induced proapoptotic signaling that differs between patients.

In most of our experiments we tested rapamycin at 100 nM. This concentration was chosen because mTOR signaling in primary AML cells is often inhibited by rapamycin 10–20 nM, but for exceptional patients there is a partial resistance, and concentrations exceeding 50 nM have to be used to achieve a reduction in downstream protein phosphorylation [18]. Concentrations up to 1000 nM have been used in previous *in vitro* studies [18], and another recent AML study also used rapamycin 100 nM [30]. In our dose-response experiments we observed that a plateau was reached when testing the effect of higher rapamycin concentrations (>10–20 nM) on cytokine-dependent AML cell proliferation, and a similar plateau was also observed in an experimental myeloma model [17]. The detection of a plateau suggests that no functionally important off-target effects occur when using rapamycin 100 nM. Finally, we used flow cytometric detection of the phosphorylated form of S6RP as a marker of mTOR signaling; this is a downstream mediator to mTOR, and previous AML studies using the alternative methodological approach with western blot analysis have shown that rapamycin will decrease S6RP phosphorylation [31] (Reikvam, submitted).

The intracellular levels of autophagy- and apoptosis-regulating mediators showed a considerable variation in primary AML cells, but the levels did not have a close association with spontaneous apoptosis or the effect of rapamycin. Furthermore, valproic acid altered the levels of several of these mediators but did not affect viability or autophagy when added together with rapamycin. Taken together these observations suggest that the intracellular levels of these mediators do not have any major role in our experimental model with regard to regulation of viability or lysosomal acidity.

We investigated the effects of double targeting of the PI3K-Akt-mTOR pathway by combining rapamycin and 3-MA, an upstream inhibitor of PI3K and also an inhibitor of autophagy [25]. We have previously done a detailed study of 3-MA effects on primary human AML cells showing that rapamycin and 3-MA may have additive proapoptotic and antiproliferative effects in primary human AML cells [32]. Our present results with AML cell lines showed that this double targeting caused an additive reduction of the viability had no effect on cell cycle distribution but caused an unexpected increase of lysosomal acidity consistent with increased autophagy. Based on our present results we therefore conclude that (i) inhibitors targeting different steps in the PI3K-Akt-mTOR pathway have different biological effects, and (ii) the effects of combined inhibition of different steps may induce new functional effects, and the final effects of such combinations not only represent a summary of the single drug effects. An advantage of dual targeting has also been suggested by another study [33].

The combination of valproic acid and ATRA is now tried as disease-stabilizing treatment in AML [34], and these two drugs may eventually be combined with cytotoxic agents [29]. Combination with rapamycin should also be considered because this drug shows limited toxicity, and serum level estimation is available [34]. Our present results show that valproic acid (but not ATRA) can alter the levels of autophagy-associated molecules; none of the drugs interfere with the mTOR inhibitory effect of rapamycin but they had no additional biological effects compared with rapamycin alone when comparing the overall results. Future studies should therefore focus on whether it is possible to define patient subsets that will benefit from treatment with these drug combinations.

NF-*κ*B inhibition is also considered as a possible therapeutic strategy in AML [35]. This therapeutic approach seems to target the leukemic stem cells [35] as well as the communication with nonleukemic neighboring cells through the local cytokine network [14]. Even though BMS-345541 did not interfere with the mTOR inhibitory effect of rapamycin, the combination of rapamycin plus NF*κ*B inhibition had divergent effects in primary AML cells compared with rapamycin alone. However, the combination caused increased apoptosis, autophagy, and G0/G1 accumulation in the KG-1a cell line, and these mechanisms may then be important for the increased antileukemic effect of this combination in certain patients. 

Inhibition of the PI3K-Akt-mTOR pathway is regarded as a possible therapeutic strategy in human AML. Based on our present results we conclude that (i) future *in vitro* pharmacological characterization of PI3K-Akt-mTOR inhibitors requires carefully standardized experimental models, (ii) even though constitutive activation of this pathway and the inhibitory effect of rapamycin on this signaling seem to be a general characteristic of primary human AML cells, the final biological effects of rapamycin seem to differ between patients, and (iii) combined targeting of different steps in this pathway should be further investigated as a possible therapeutic strategy whereas combination of rapamycin with valproic acid, ATRA, or NF-*κ*B inhibitors seems less promising.

## Supplementary Material

Supplementary Figure 1: Effects of *in vitro* culture of primary human AML cells on viability, lysosomal acidity and mTOR signaling for primary human AML cells derived from unselected patients. (A) Leukemic cells were incubated in FBS containing medium, and the figure compares the lysosomal acidity of the cells when analyzed after 4 and 24 hours of culture. (B) The effect of serum deprivation; the figure compares mTOR mediated signaling (levels of the downstream target phosphorylated S6RP) for AML cells incubated for 24 hours in serum-free medium or medium supplemented with 10% heat-inactivated FBS. (C) The effect of culture time on lysosomal acidity for cells cultured in serum-free medium; the figure compares the acidity for cells cultured for 4 and 24 hours. (D) Lysosomal acidity for primary human AML cells cultured for 24 hours either in serum-free or FBS-containing medium.
Supplementary Figure 2: The effect of rapamycin on the viability of the AML cell lines HEL (upper) and CTV-1 (lower). Cells were cultured either in serum-free (right part) or FBS-supplemented medium (left part) for 24 hours before the viability was analyzed by flow cytometry. In all the cultures we identified viable Annexin-V^−^PI^−^, early apoptotic Annexin-V^+^PI^−^ and late apoptotic/necrotic Annexin-V^+^PI^+^ cells, whereas Annexin-V^−^PI^+^ necrotic cells could not be detected. Rapamycin was tested at 100, 200, 400, 600 and 800 nM. The figure presents the percentage of viable, apoptotic and late apoptotic/necrotic cells.
Supplementary Figure 3: The effect of FBS supplementation on lysosomal acidity of the AML cell lines HL60, HEL, KG1a, CTLV-1 and K562 cultured with and without FBS. The cells were cultured for 4 hours in medium with (+FBS) and without (-FBS) heat-inactivated FBS before flow cytometric analysis of lysosomal acidity. We examined the cell lines with (right part) and without (left part) rapamycin 100 nM as indicated at the top of the figure. The figure shows the diagrams for the flow-cytometric analyses for each of the cell lines and the MFI for each analysis is given in the figure. These data suggest that the increased lysosomal acidity during serum deprivation is not dependent on mTOR signaling.
