# Supplementary material for: Inhibition of Mammalian Target of Rapamycin in Human Acute Myeloid Leukemia Cells Has Diverse Effects That Depend on the Environmental In Vitro Stress
Source: Bone Marrow Res. 2012 Oct 2;2012:329061. doi: 10.1155/2012/329061 (PMC3467767; doi:10.1155/2012/329061)
Supplement: Supplementary file 1 — Supplementary Figure 1: Effects of in vitro culture of primary human AML cells on viability, lysosomal acidity and mTOR signaling for primary human AML cells derived from unselected patients. (A) Leukemic cells were incubated in FBS containing medium, and the figure compares the lysosomal acidity of the cells when analyzed after 4 and 24 hours of culture. (B) The effect of serum deprivation; the figure compares mTOR mediated signaling (levels of the downstream target phosphorylated S6RP) for AML cells incubated for 24 hours in serum-free medium or medium supplemented with 10% heat-inactivated FBS. (C) The effect of culture time on lysosomal acidity for cells cultured in serum-free medium; the figure compares the acidity for cells cultured for 4 and 24 hours. (D) Lysosomal acidity for primary human AML cells cultured for 24 hours either in serum-free or FBS-containing medium. Supplementary Figure 2: The effect of rapamycin on the viability of the AML cell lines HEL (upper) and CTV-1 (lower). Cells were cultured either in serum-free (right part) or FBS-supplemented medium (left part) for 24 hours before the viability was analyzed by flow cytometry. In all the cultures we identified viable Annexin-V−PI−, early apoptotic Annexin-V+PI− and late apoptotic/necrotic Annexin-V+PI+ cells, whereas Annexin-V−PI+ necrotic cells could not be detected. Rapamycin was tested at 100, 200, 400, 600 and 800 nM. The figure presents the percentage of viable, apoptotic and late apoptotic/necrotic cells. Supplementary Figure 3: The effect of FBS supplementation on lysosomal acidity of the AML cell lines HL60, HEL, KG1a, CTLV-1 and K562 cultured with and without FBS. The cells were cultured for 4 hours in medium with (+FBS) and without (-FBS) heat-inactivated FBS before flow cytometric analysis of lysosomal acidity. We examined the cell lines with (right part) and without (left part) rapamycin 100 nM as indicated at the top of the figure. The figure shows the diagrams for the flow-cytometri [file 329061.f1.pdf]

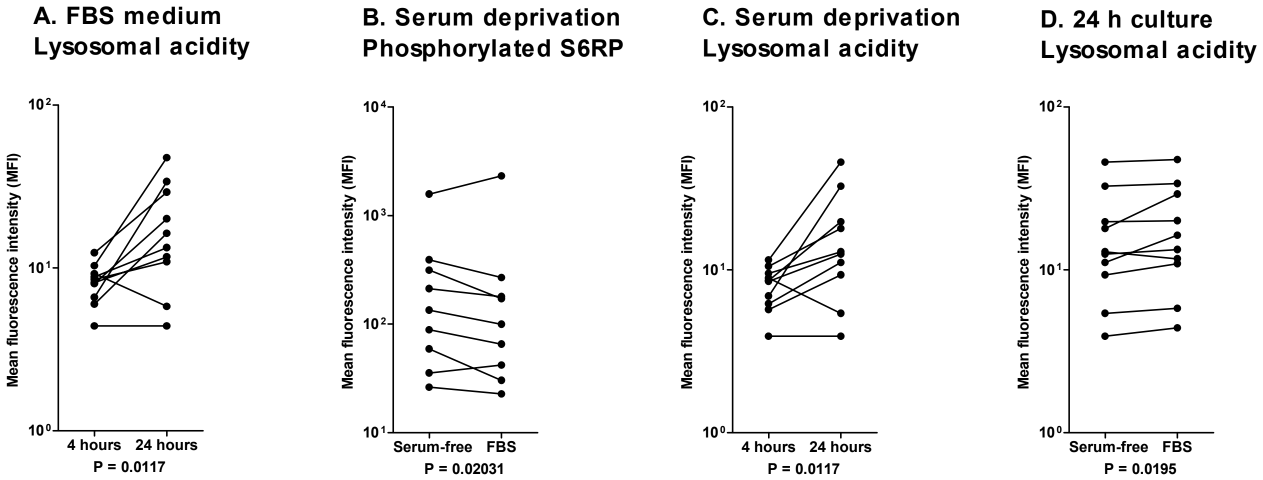
A. FBS medium
Lysosomal acidity
B. Serum deprivation
Phosphorylated S6RP
C. Serum deprivation
Lysosomal acidity
D. 24 h culture
Lysosomal acidity
Mean fluorescence intensity (MFI)
10^2
10^1
10^0
10^4
10^3
4 hours
24 hours
Serum-free
FBS
P = 0.0117
P = 0.02031
P = 0.0117
P = 0.0195
