# Supplementary figures and images for: Inhibition of Mammalian Target of Rapamycin in Human Acute Myeloid Leukemia Cells Has Diverse Effects That Depend on the Environmental In Vitro Stress
Source: Bone Marrow Res. 2012 Oct 2;2012:329061. doi: 10.1155/2012/329061 (PMC3467767; doi:10.1155/2012/329061)

**+ FBS****- FBS****HEL**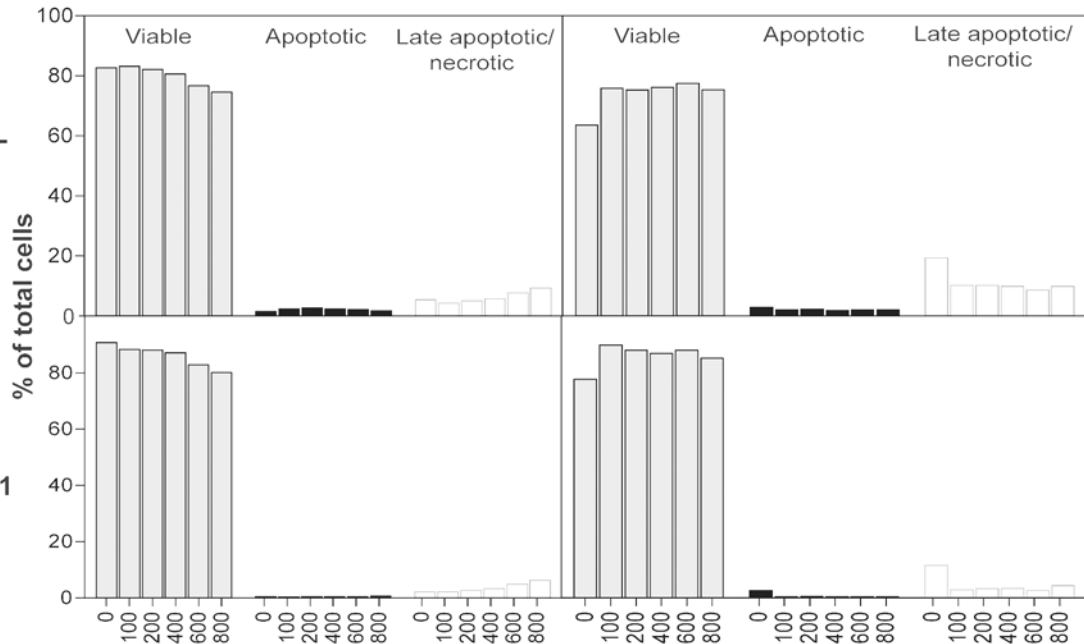

Supplement: Supplementary file 2 [file 329061.f2.pdf]
