# Supplementary material for: Inhibition of Mammalian Target of Rapamycin in Human Acute Myeloid Leukemia Cells Has Diverse Effects That Depend on the Environmental In Vitro Stress
Source: Bone Marrow Res. 2012 Oct 2;2012:329061. doi: 10.1155/2012/329061 (PMC3467767; doi:10.1155/2012/329061)
Supplement: Supplementary file 3 [file 329061.f3.pdf]

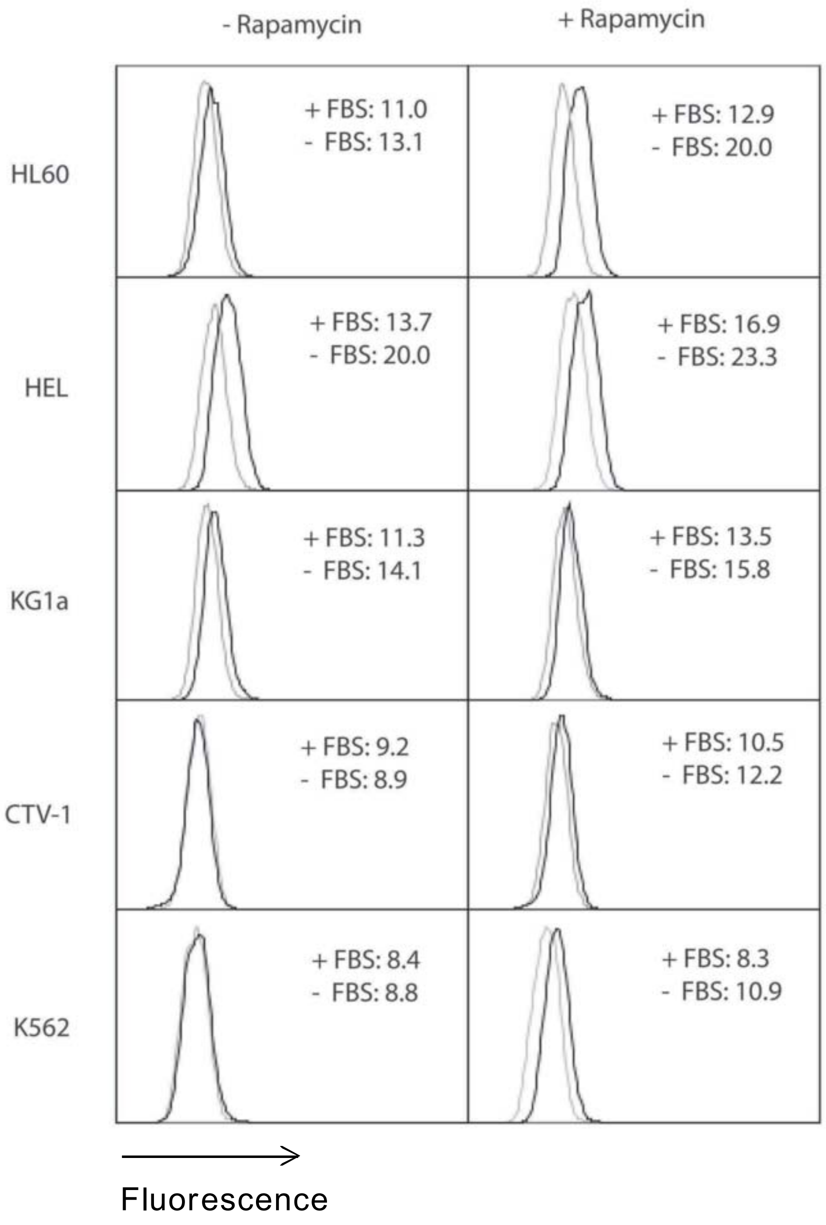

- Rapamycin
+ Rapamycin
HL60
+ FBS: 11.0
- FBS: 13.1
+ FBS: 12.9
- FBS: 20.0
HEL
+ FBS: 13.7
- FBS: 20.0
+ FBS: 16.9
- FBS: 23.3
KG1a
+ FBS: 11.3
- FBS: 14.1
+ FBS: 13.5
- FBS: 15.8
CTV-1
+ FBS: 9.2
- FBS: 8.9
+ FBS: 10.5
- FBS: 12.2
K562
+ FBS: 8.4
- FBS: 8.8
+ FBS: 8.3
- FBS: 10.9
Fluorescence
